# Building human renal tracts

**DOI:** 10.1016/j.jpedsurg.2021.10.022

**Published:** 2022-02

**Authors:** Adrian S. Woolf

**Affiliations:** a Division of Cell Matrix Biology and Regenerative Medicine, School of Biological Sciences, Michael Smith Building, Faculty of Biology Medicine and Health, University of Manchester, Oxford Road, Manchester, Northern Ireland M13 9PT, United Kingdom; b Royal Manchester Children's Hospital, Manchester University NHS Foundation Trust, Manchester Academic Health Science Center, Manchester, Northern Ireland, United Kingdom

**Keywords:** Nephron, Collecting duct, Implant, Pluripotent stem cell, Ureter

## Abstract

Severe kidney failure affects several million people worldwide. Among these are children born with abnormal renal tracts, and some carry mutations of genes active in renal tract development. Kidney transplants are in short supply, and long term dialysis does not obviate uraemia and its associated harmful effects. It has been envisaged that a combination of stem cell technology, developmental biology, and genetics will revolutionise our understanding of kidney disease and provide novel therapies for kidney failure. Here, we review progress towards making functional kidney tissues from human pluripotent stem cells. Organoids rich in immature glomeruli and tubules can be created in culture from pluripotent stem cells. Moreover, differentiation can be increased by implanting these cells into immunodeficient mice. Challenges remain to be overcome, however, before these tissues can be used for regenerative medicine therapies. Current limitations include the small size of an organoid, the lack of large blood vessels feeding it, and the lack of a urinary tract to plumb the kidney organoid. Pluripotent stem cell technology is also being used to create ‘diseases in a dish’ to understand the pathobiology underlying human renal tract malformations.

## Highlights

• Human pluripotent stem cells can be differentiated in culture to form organoids containing immature nephrons and collecting ducts.
• After implantation into immunodeficient mice, kidney precursor cells derived from human pluripotent stem cells show enhanced differentiation, with the formation of glomeruli containing capillary loops.
• Use of organoids for regenerative medicine therapies is currently precluded by their small size, the lack of a large artery feeding the organoid, and the lack of a urinary tract to plumb the kidney organoid.
• While a whole ureter has yet to be made from pluripotent stem cells, the latter can be differentiated into urothelial cells.
• The ability to create ‘kidney diseases in a dish’ from human pluripotent stem cells is beginning to provide insights into the pathobiology of congenital renal diseases, especially those caused by mutations.

## 1 Introduction

Worldwide around 2.8 million individuals receive renal replacement therapy (RRT) for end stage kidney failure (ESKF) [1]. It has been estimated that at least two million further individuals need transplantation or long-term dialysis to survive but are unable to access these therapies [1]. Moreover, even in high-income developed countries, transplants are in short supply, and life expectancy on long term dialysis is severely curtailed compared with life expectancy in the average aged matched population [2]. Based on surveys, there are around 8000 children in Europe and 9000 children in the US treated with RRT, with an estimated worldwide prevalence of 150,000 [3,4]. With regard to young children with ESKF, around half were born with abnormal renal tracts i.e. with kidney malformations, often accompanied urinary tract (ureter, bladder and urethra) anomalies [3]. Moreover, it has been estimated that up to one fifth of young adults with ESKF were born with renal tract malformations [5].

Given the above information, there are pressing needs to discover new therapies with which to either treat ESKF or to slow the progressive loss of kidney excretory function that generally precedes ESKF. It is in these contexts that human pluripotent stem cell (PSC) technology offers promise. Here, we review progress towards making functional kidney tissues from human PSCs. The substantial challenges to achieving this end will be discussed. We also indicate how human PSCs are being used to create ‘diseases in a dish’ to understand the pathobiology underlying congenital kidney disease.

### 1.1 Introduction to PSCs and kidney development

**Fig. 1:**
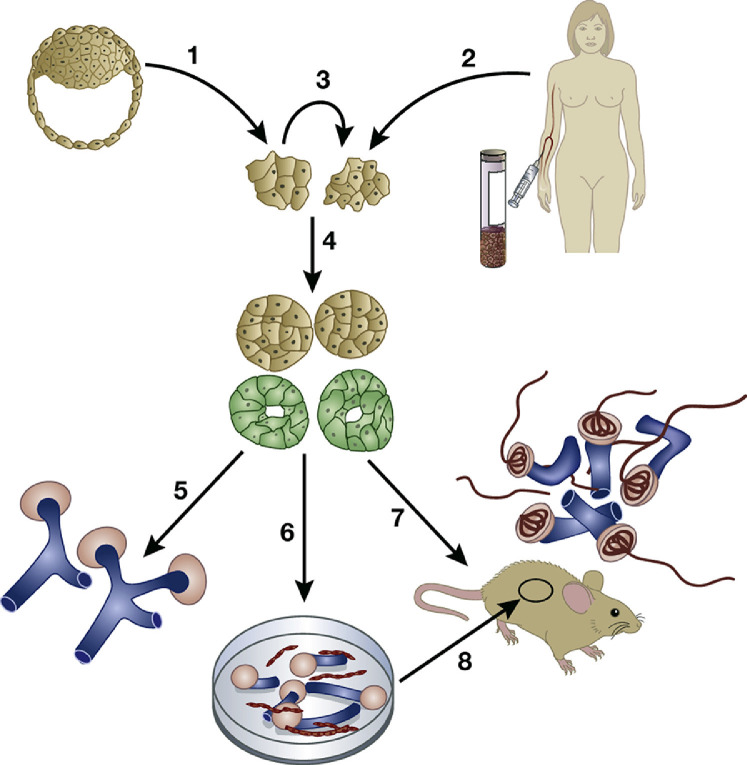
Using human pluripotent stem cell (PSC) technology to generate kidney tissues. Human PSCs can be made from the inner cell mass of early human embryos (arrow 1) or by generating induced PSCs from mature cells including those in the blood, skin, or shed into the urine (arrow 2). PSCs can undergo self renewal (arrow 3), or they can be induced by application of growth factors and other molecules to differentiate in culture (arrow 4) into intermediate mesoderm like cells that then begin to express molecules (green) found in the developing kidney. These kidney precursor cells can be maintained in 2 dimensional culture (arrow 5) where they form tubule like structures (blue) and primitive nephrons (pink). Alternatively, PSC derived kidney precursor cells can be dissociated and plated in 3 dimensional masses (arrow 6) that differentiate to form tubules (blue) and avascular glomeruli (pink). These organoids contain endothelia (red) between tubules but they do not invade glomerular tufts. A third option is to implant PSC derived kidney precursor cells into immune deficient mice (arrow 7), where the human cells form vascularized glomeruli (red inside pink structures). A similar result can be obtained after implanting PSC derived kidney organoids under the kidney capsule inside immune deficient mice (arrow 8). Image is from [6]. Woolf AS. Growing a new human kidney. Kidney Int. 2019;96:871–882 via Creative Commons Attribution License (CC BY).

By definition, PSCs have the ability to replicate themselves in perpetuity. In addition, they have the potential to differentiate into the three germ layers (mesoderm, endoderm and ectoderm) of the early embryo and thus form all organs. As recently reviewed [6,7] and depicted in Fig. 1, there are two sources of PSCs for research studies. The first is the inner cell mass of very early human embryos that have been generated, but eventually not placed in the uterus, during *in vitro* fertilization therapy. This type of PSC is called an embryonic SC (ESC). More recently, it has become possible to make ‘induced’ PSC (iPSCs) by taking a sample of differentiated cells (e.g. white blood cells, skin fibroblasts or even renal cells shed into the urine) and driving these into PSCs by viral vector mediated transient expression of pluripotency molecules.

Given the ability to source human PSCs, the question is how to drive these to become kidney tissues in the laboratory. Here, stem cell researchers have been much informed by classical studies of the anatomy and developmental biology of kidney development. Over half a century ago, Edith Potter made detailed morphological and histological descriptions of normal human embryonic and fetalkidneys, from their inception at five weeks gestation to the end of their development in the late third trimester [8,9]. She also documented the perturbed anatomy of human dysplastic kidneys, either arising from primary disturbances in the ureteric bud (UB) and metanephric mesenchyme (MM) compartments, or arising from the physical disruption of impairment of fetalurinary flow caused by urinary tract obstruction [8,9].

**Fig. 2:**
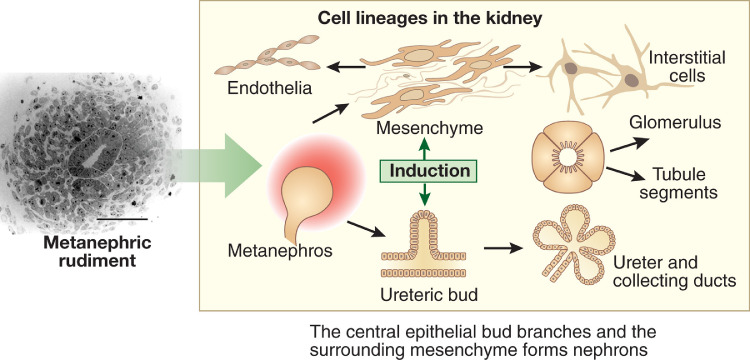
Cell lineages in the embryonic kidney. The frame on the left shows histology of the metanephros at its inception, with a central ureteric bud surrounded by metanephric mesenchyme. Bar = 50 μm. The frame on the right depicts mutual induction between these compartments. The ureteric bud differentiates into the urothelial stalk of the ureter and the arborizing collecting ducts within the kidney. The metanephric mesenchyme undergoes mesenchymal to epithelial transition to form nephrons, comprising glomerular and tubule epithelia, whereas other cells in the mesenchymal compartment will form interstitial cells and endothelia [6]. Woolf AS. Growing a new human kidney. Kidney Int. 2019;96:871–882 via Creative Commons Attribution License (CC BY).

A second line of studies were initiated at a similar time by Clifford Grobstein [10] and then continued by Saxen and Thesleff other pioneers [11]. These researchers showed that embryonic mouse kidney rudiments could be explanted into organ culture and maintained for around a week during which time the UB elongated into a ureteric stalk, the top of which branched recurrently to form collecting ducts, and some MM cells underwent a mesenchymal to epithelial transition to form nephrons (glomerular podocytes and Bowman capsule, proximal tubules and distal tubules), as depicted in Fig. 2. Other MM cells differentiate into interstitial cells located between the tubules and at least a subset of kidney capillaries [12]. Conversely, if the UB or MM was cultured in isolation, each component failed to differentiate and instead died by apoptosis.

Subsequent experiments using organ culture or wild type murine tissues and also mutant mice [13] showed that the MM and UB nurtured each other, a process called mutual induction (Fig. 2), by secreting a variety of paracrine growth factors. Moreover, other types of molecule, especially transcription factors and extracellular matrix proteins, were found to be expressed by the developing kidney and were essential for its normal development [13]. Of note, the genes encoding several of these molecules have been found to be mutated in individuals with congenital kidney disease [7]. The UB and MM themselves each originate from separate compartments within the intermediate mesoderm [14], that itself has formed during gastulation, the name of the event when the three germ layers form early on in embryogenesis. This gives insight into growth factors and other molecules that, during normal development, generate the building blocks of the kidney rudiment [14].

### 1.2 Making kidney like tissues from PSCs

In the last decade, as reviewed [6,7], several laboratories around the world have published protocols in which human PSC can be driven by sequential addition of specific growth factors and other chemicals to form kidney like tissues in 2D and in 3D, or organoid, culture (Fig. 1). During these protocols, PSCs are differentiated to mesodermal precursors of either MM or UB cells, and then these precursors are given further stimulation to become nephrons [15,16] or collecting ducts [14,17]. In general, the protocols generate just one of these two kidney lineages, as demonstrated by single cell RNA sequencing to interrogate the molecular phenotypes of individual cells within organoids [15]. There does, however, appear to be some plasticity in these systems and, for example, nephron distal tubule precursors can be reprogrammed in culture to transdifferentate into collecting ducts [16]. We have experience with the nephron culture protocol and, like other investigators, have demonstrated that the resulting organoids contain glomeruli, proximal tubules and distal tubules [18,^19^].

A limitation, however, is that the resulting glomeruli contain only epithelial cells (podocytes and the Bowman capsule) and lack capillary loops and mesangial cells in their tufts [18,19]. This is despite the fact that capillaries are detected between tubules in the organoid. Clearly, avascular glomeruli have no chance of filtering blood to make urine and, moreover, the organoids anyhow lack a blood supply. We had previously documented that, after native embryonic mouse kidney rudiments were transplanted into the nephrogenic cortex of newborn mice, the transplant differentiated into glomeruli containing capillary loops [12]. Furthermore, after fluorescently labelled low molecular dextran was intravenously injected into the host mouse, we detected fluorescent signal in proximal tubules that had formed in the transplant [20]. This was consistent with the occurrence of ultrafiltration by glomeruli followed by reuptake by tubules.

**Fig. 3:**
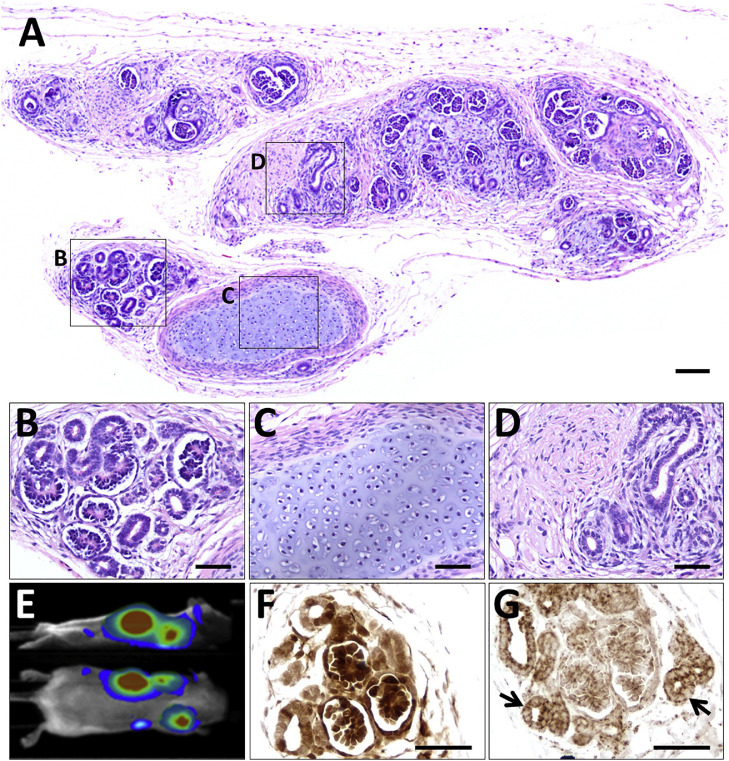
Tissues harvested at 12 weeks after subcutaneous implantation into immunodeficient mice of luciferase labeled human PSC derived kidney precursor cells. (A–D) Histology overview 12. The implanted cells have formed a differentiated mass (A). Boxed areas indicate: (B) relatively well differentiated nephrons and tubules; (C) cartilage, an off target mesodermal derived tissue; and (D) poorly differentiated tubules and stromal cells. (E) Side and dorsal views showing bioluminescence in a living mouse that had received kidney precursor implants; the mouse had been administered luciferin, the substrate for luciferase. (F) Immunostaining (brown) for luciferase in area containing nephron like structures. (G) Immunostaining (brown) for human mitochondria, arrows indicate tubules.Sections in (F) and (G) are not counterstained. Scale bars, 100 μm (A) and 50 μm in (B)–(D), (F), and (G). Images are from [18] Bantounas et al. Generation of functioning nephrons by implanting human pluripotent stem cell derived kidney progenitors. Stem Cell Reports. 2018;10:766–779 via Creative Commons Attribution License (CC BY).

With these results in mind, we reasoned that implanting human PSC derived kidney precursor cells subcutaneously into immunocompromised mice would lead to the creation of more structurally mature and functional nephrons. As depicted in Fig. 3, 12 weeks after implantation, we were able to visualise living implants, the PSC having been molecularly labelled with a luciferase expressing transgene. On histology, the implanted cells had formed structures up to around 1 cm in length and they contained glomeruli and tubules. The former contained capillary loops and mesangial like cells. Not all parts of the implant were so mature and these other areas contained thin tubules surrounded by stromal cells; moreover, some implants contained islands of cartilage like cells, ‘off target’ mesodermal derivatives (Fig. 3). It was also demonstrated that, after injection of fluorescently labelled dextran into the host, a subset of tubules in the implant contained florescent granules, suggestive of endocytosed ultrafiltrate [18]. Other investigators were able to show that, after implantation of human PSC derived organoids under the kidney capsule of host mice, the arising glomeruli also contain capillary loops [21].

### 1.3 Remaining limitations to creating functional renal tracts from PSCs

**Fig. 4:**
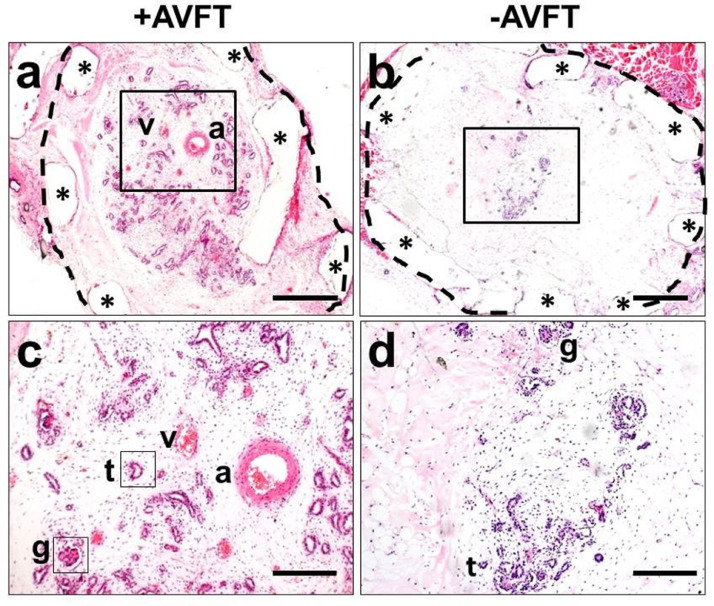
Identification of kidney like tissues within implanted chambers seeded with human PSC derived kidney precursor cells. a–d. Histology sections stained with hematoxylin and eosin of chambers harvested three weeks after surgery. Bars are 500 μm in (a, b) and 200 μm in (c, d). a Low power of cross section of chamber with an arterio venous flow through (+AVFT). A dotted line has been drawn around the outside of the chamber and asterisks indicate the chamber walls. *a* and *v* indicate the host femoral artery and vein, respectively. The kidney like structures appear blue and an enlargement of the boxed area is depicted in *c*, where tubule like (t) and glomerular-like (g) structures separated by loose stromal like tissues are noted. b,and d respectively show an overview and an enlarged section of a chamber lacking an arterio venous flow through ( AVFT). Note that this chamber contains a few tubules but, as expected, lacks a large artery or vein. Images from [22] Ranjzad et al. Aberrant differentiation of human pluripotent stem cell derived kidney precursor cells inside mouse vascularized bioreactors. Nephron. 2020;144:509–524 via Creative Commons Attribution License (CC BY).

Although the above advances in organoid culture and after implantation are encouraging, it is worth reflecting on the remaining challenges before clinically useful functional intact renal tracts might be generated from human PSCs. As reviewed [6], the first problem is one of scale: an organoid might contain up to around 100 glomeruli but a healthy human kidney contains over three magnitudes more. Second, although organoid implants are perfused by host blood, there are only small calibre connecting vessels. Presumably, given that glomerular filtration is positively correlated with blood flow and hydrostatic pressure, without a more robust arterial supply implant glomeruli would never generate much urine. With this in mind, we undertook experiments in which human PSC derived kidney precursor cells were paced inside perforated plastic chambers which we implanted into the thighs of immunocompromised mice [22]. In some implants we surgically fashioned an ‘arterio venous flow through’ in which the host's femoral artery and vein were mobilized and threaded through the chamber. The rationale here was that these might enhance the vascularity and growth of the implanted cells, as had been described for other, non renal, cell implants [23]. By comparing histology of kidney precursor implants with or without flow throughs, it was demonstrated that the former enhanced the number of small vessels around the human implants at three weeks after surgery. While kidney like tissues were seen in all implants (Fig. 4), quantification did not reveal a significant difference between the two conditions. Moreover, at 12 weeks after surgery, we documented an overabundance of scar like interstitial tissues of human origin in implants with an arterio venous flow through [22], suggesting that pathological differentiation might follow a period of increased vascularity.

**Fig. 5:**
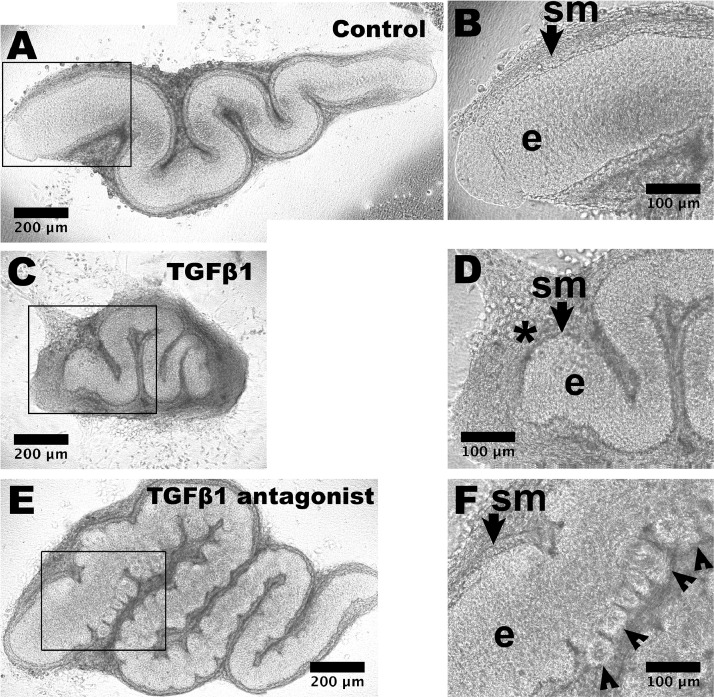
Manipulating the growth of explanted embryonic ureters with growth factors. Embryonic day 15 mouse ureters were viewed as whole mounts at day 6 of organ culture. (A) An organ fed basal media only shows a snake like appearance, with high power of the proximal part (boxed) shown in (B). The SM layer (sm, arrowed) and epithelium (e) are indicated. (C) An organ fed with media supplemented with TGFβ1 is smaller than the control organ. In the high power view (D), the ureter tube is surrounded by a prominent interstitial cell layer (asterisk). (E) Organ after 6 days of culture fed with media supplemented with 10 μm LY2109761 that inhibits TGFβRI/TGFβRII kinase. Note the overgrown appearance compared with the control organ, with numerous bud like structures protruding from the epithelial tube. Some of these are visualized (arrowheads) in the high power image (F). Images from [30] Lopes et al. Overactivity or blockade of transforming growth factor β each generate a specific ureter malformation. J Pathol. 2019;249:472–484 via Creative Commons Attribution License (CC BY).

A third challenge is that human PSC derived kidney nephron generated in culture or after implantation lack a ureter. Without such a drainage tube, any urine generated by the implant would simply diffuse back into the host. Efforts are underway to generate ureteric tissues from PSCs, with reports of success in driving PSCs to become epithelia with a urothelial phenotype [24], [25], [26]. These models, however, do not appear to generate ureteric smooth muscle cells. Without proximal to distal directional peristalsis, that initiates before birth in native ureters, there would be the risk of ‘functional obstruction to urine flow’ as occurs in *Teashirt 3* mutant mice that lack ureteric smooth muscle yet have an anatomically patent urothelial tube [27]. In normal organogenesis, immature urothelia act as a paracrine signalling center, secreting the sonic hedgehog growth factor that induces nearby mesenchymal cells to form smooth muscle [28,29]. Of note, when PSC derived urothelia were implanted next to similar native mesenchymal cells in explant culture, the latter were induced to differentiate into contractile muscle [26]. As for kidney organoids generated from PSCs, ureteric tissues derived from PSCs are smaller than their *in vivo* native counterparts. It is established that the linear growth of explanted mouse embryonic ureters can be inhibited by application of exogenous transforming growth factor β (TGFβ) or accelerated by blockade of endogenous TGFβ signalling (Fig. 5) [30]. Moreover, application of exogenous fibroblast growth factor 10 (FGF10) stimulated linear growth [30]. Such observations may inform manipulations of PSC derived urothelial tissues to increase their size. Indeed, it has been reported that application of FGF10 to PSC derived bladder like urothelia enhanced their molecular maturity and stratification [25].

### 1.4 Modeling human congenital kidney disease with human PSC technology

As discussed in the *Introduction* to review, congenital disorders of development and differentiation of the kidney and urinary tract can cause ESKD. In the last two decades, it has become apparent that at least a small subset of individuals both with kidney [7,31] or urinary tract [32] malformations carry mutations of genes active during normal renal tract development. At the time of writing, the commonest mutated gene associated with cystic dysplastic kidneys is hepatocyte *nuclear factor 1B* (*HNF1B*); for example, pathogenic heterozygous variants of *HNF1B* were reported in 52 of 199 children with kidney malformations [33]. This gene codes for a transcription factor expressed in tubules derived from both the UB and the MM [34]. It is rare to obtain tissue samples of these native kidneys and researchers are turning PSC organoid technology to try to model the disease. One research group [35] used CRISPR Cas9 gene editing to mutate one allele of *HNF1B* in PSCs. After driving these to become UB like tissues, they recorded impaired branching into collecting duct precursors. Moreover, these primitive ducts showed aberrant expression of several molecular markers of the UC/collecting duct lineage [35]. Another research group mutated both alleles of *HNF1B* in human PSC and then drove them to become nephron organoids [36]. They reported that the mutant organoids contained glomeruli but appeared to lack proximal and distal tubules. A caveat with this PSC study is that their model had both copies of the gene mutated whereas humans have only been reported with one copy mutated, with dominant inheritance of the disease through several generations.

The interested reader is directed to a recent review that addresses the use of human PSCs to study a wide range of inherited human kidney diseases including the polycystic kidney diseases and congenital nephrotic syndrome [7]. In the longer term it is hoped that mechanistic insights from such studies will suggest druggable targets to enhance differentiation and preclude or at least delay the onset of ERKF.

## 2 Conclusion

Human PSC technology has made striking advances in generating kidney like tissues. The potential use of organoids for regenerative medicine therapies is currently precluded by their small size, the lack of large vessels feeding blood to the organoid, and the lack of a urinary tract to drain urine made by the newly grown kidney. The ability to model kidney diseases starting with human PSC should allow insights into the pathobiology of congenital renal diseases, especially those caused by mutations.

## Declaration of Competing Interest

The author declared no competing interests.
